# B-Cell-Activating Factor Affects the Occurrence of Thyroid Autoimmunity in Chronic Hepatitis C Patients Treated with Interferon Alpha

**DOI:** 10.1155/2012/247973

**Published:** 2012-02-27

**Authors:** Yusuke Kajiyama, Kentaro Kikuchi, Atsuko Takai, Naomi Hosoya, Hiromi Hoshino, Kunihiko Hino, Hiroshi Miyakawa

**Affiliations:** ^1^Fourth Department of Internal Medicine, Teikyo University Mizonokuchi Hospital, 3-8-3 Mizonokuchi, Kawasaki-shi, Kanagawa 213-8507, Japan; ^2^Central Laboratory, Teikyo University Mizonokuchi Hospital, 3-8-3 Mizonokuchi, Kawasaki-shi, Kanagawa 213-8507, Japan; ^3^Delta Clinic, 2-5-1 Kusunokidai, Tokorozawa-shi, Saitama 359-0037, Japan

## Abstract

Chronic hepatitis C (CHC) patients frequently suffer from thyroid disorders during interferon therapy. However, the mechanism remains unclear. In this study, we investigated the association between serum B-cell-activating factor belonging to the TNF family (BAFF) levels and the presence of antithyroid peroxidase antibody (anti-TPO) in CHC patients treated with pegylated interferon alpha and ribavirin combination therapy. Six months after the therapy, anti-TPO antibody was detected in 10 (males, 1; females, 9) of 50 patients. The mean age of these patients was higher than that of the anti-TPO-negative patients (61 yr versus 55 yr). Before treatment, the serum BAFF levels of the anti-TPO-positive patients were higher than those of the anti-TPO-negative patients. After starting therapy, the serum BAFF levels of both the anti-TPO-positive and -negative patient groups were elevated. Our findings suggest that the serum BAFF concentration before therapy can predict the risk of thyroid autoimmunity in elderly female patients with CHC.

## 1. Introduction

Interferon alpha (IFN*α*) is a type I interferon that has been widely used as a therapeutic agent, mostly for infectious diseases, including chronic hepatitis C virus (HCV) infection [1]. IFN*α* therapy is associated with many side effects such as flu-like symptoms, hematologic disorders, and neuropsychiatric disorders [2]. One of the commonest side effects of IFN*α* therapy is autoimmune thyroid disorders manifesting as Hashimoto's thyroiditis, Graves disease, or the production of thyroid autoantibodies without any thyroid dysfunction [3–5]. However, the detailed mechanism of these effects is unknown.

B-cell-activating factor belonging to the TNF family (BAFF), which is also known as BLyS, TALL-1, zTNF4, or THANK, is part of the TNF family and is known to play an important role in the differentiation of B cells and the maintenance of mature B-cell shape [6–10]. BAFF is expressed on the surfaces of monocytes, dendritic cells, neutrophils, activated T cells, malignant B cells, and epithelial cells [6–10]. BAFF plays an important role in humoral immunity.

The N-terminal sequence of human BAFF contains a furin cleavage site that is responsible for the release of soluble BAFF [8]. After the development of ELISA using monoclonal antibody, high concentrations of BAFF were clinically measured in patients with autoimmune diseases such as rheumatoid arthritis, autoimmune diabetes, Sjögren's syndrome, and multiple sclerosis [11–15]. It was further found that BAFF affects the regulation of the interaction between antigen-presenting cells and T cells, resulting in the emergence of several autoantibodies [16].

So, in the present study, to address the onset mechanism of IFN-induced thyroid autoimmunity, we investigated anti-TPO antibodies and serum BAFF levels in chronic hepatitis C (CHC) patients treated with IFN*α*.

## 2. Materials and Methods

### 2.1. Patients

Fifty CHC patients (males, 24; females, 26) who received pegylated interferon alpha (PEG-IFN*α* 2b) and ribavirin therapy were enrolled in this study. Their mean age was 57.0 ± 7.1 years old. All patients were diagnosed with chronic hepatitis based on liver pathological findings and were positive for serum HCV RNA before therapy. After obtaining written informed consent, venous blood was collected by venipuncture and was permitted to clot. Serum samples were collected and stored at −70°C.

Serum anti-TPO antibody, TSH, and free T4 levels were examined prior to therapy and six months after the start of therapy using commercially available ECLIA kits (MBL, Nagoya, Japan and Eiken, Tokyo, Japan). The standard values of anti-TPO, TSH, and free T4 are less than 16 IU/mL, 0.5–5.0 *μ*IU/mL, and 0.9–1.7 ng/dL, respectively. Prior to therapy, all patients were confirmed to be negative for anti-TPO and to be within normal limits for TSH and free T4.

### 2.2. Serum BAFF Concentration

The serum BAFF concentration was examined by a commercially available sandwich ELISA, the Quantikine Human BAFF/BLyS/TNFSF13B Immunoassay (R&D Systems, MI, USA), using monoclonal antibody specific to BAFF [11]. All of the subjects' serum samples were assayed on the same day. The standard serum BAFF value was set from sera of 72 healthy control subjects (males, 35; females, 37. mean age, 56.3 ± 6.2 y.o.).

### 2.3. Statistical Analysis

Data are expressed as the mean ± standard deviation (SD), and all analyses were performed using the nonparametric Mann-Whitney test and chi-square test. We considered *P* values of <0.05 to be significant.

## 3. Results

### 3.1. Anti-TPO Antibody, TSH, and Free T4 Levels

**Figure 1:**
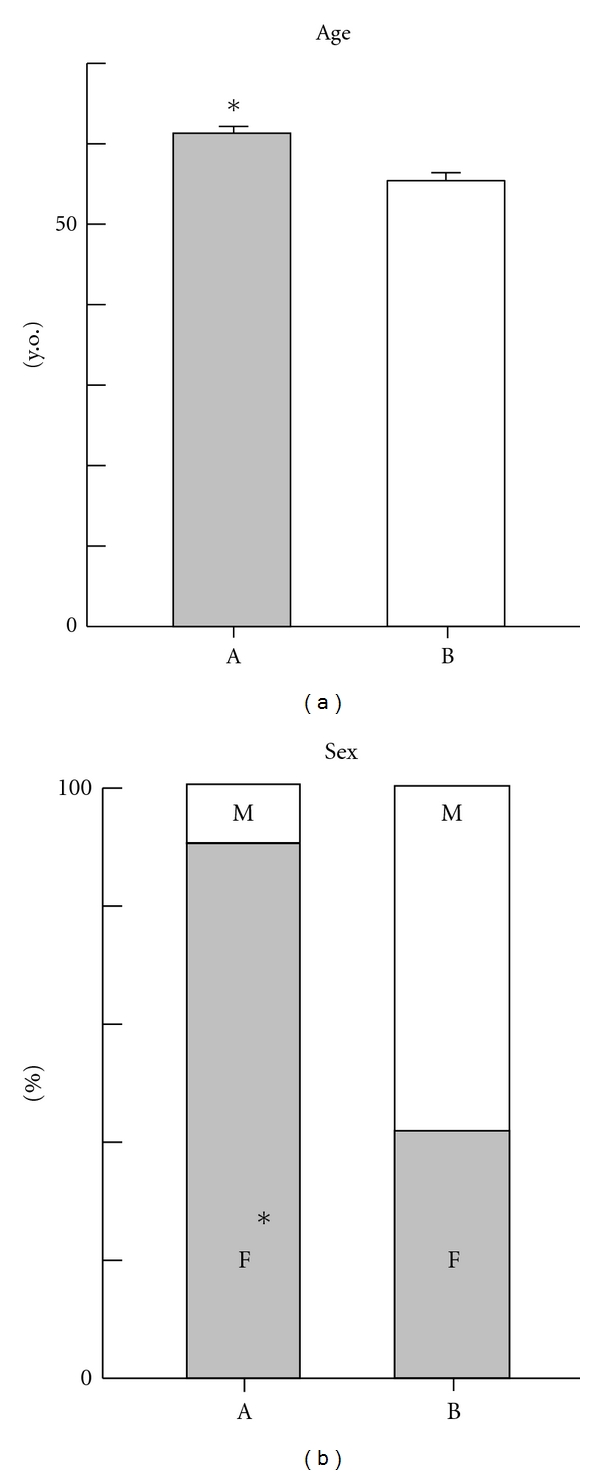
Clinical features (age, sex) of CHC patients. (a) patients who developed anti-TPO antibodies (*n* = 10) at six months after the start of peg-interferon and ribavirin therapy. (b) Patients who not developed anti-TPO antibodies (*n* = 40) at six months after the start of peg-interferon and ribavirin therapy. *P < 0.05 was statistically significant.

Before therapy, all study patients were negative for anti-TPO antibody. Six months after the start of treatment, anti-TPO antibodies were newly detected in 10 (20%) of 50 patients. Hereafter, the 10 patients in whom anti-TPO antibody was detected six months after the start of IFN therapy are referred to as group A and the other 40 patients are referred to as group B. As shown in Figure 1, the mean age of the group A patients (61.2 ± 3.8 y.o.) was significantly higher than that of group B (55.6 ± 7.9 y.o.) (*P* = 0.03). The female-to-male ratio of group A was 90% (males, 1; females, 9), and that of group B was 42.5% (males, 23; females, 17). The difference between the two groups was significant (*P* = 0.001). In group A, the mean TSH level before therapy was 1.7 ± 0.6 *μ*IU/mL, and that at six months after the start of IFN therapy was 2.1 ± 1.4 *μ*IU/mL. The mean free T4 level before therapy was 1.1 ± 0.1 ng/mL, and that at six months after the start of IFN therapy was 1.3 ± 0.3 ng/mL. There was no significant difference between the two groups. In addition, the sustained virological response rate in group A was 60% (6/10), and that in group B was 50% (20/40), which were not significantly different.

### 3.2. Serum BAFF Levels

**Figure 2:**
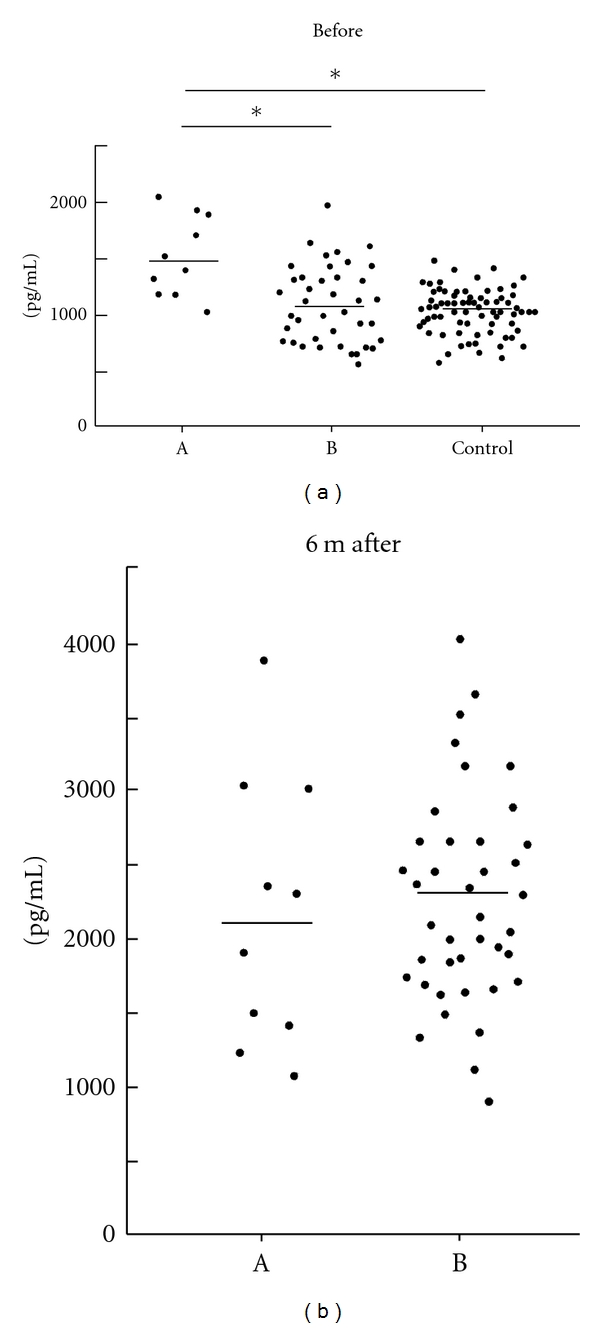
Serum BAFF concentrations of (a, b) (before treatment, at 6 months after the start of therapy) and healthy control subjects. *P < 0.05 was statistically significant.

As shown in Figure 2, the mean serum BAFF level prior to IFN therapy in group A (1497.4 ± 319.4 pg/mL) was significantly higher than that in group B (1139.5 ± 359.1 pg/mL) and healthy control subjects (1105.0 ± 215.2 pg/mL) (*P* < 0.05). The mean serum BAFF levels of both A and B groups were higher at six months after the start of IFN therapy than before therapy; however, they were not significantly different (group A: 2177.8 ± 753.3 pg/mL, group B: 2302.3 ± 660.6 pg/mL).

## 4. Discussion

Recent well-controlled studies demonstrated that both hypothyroidism and thyroid autoimmunity were significantly more common in patients with CHC than in the control population [17, 18]. Moreover, in CHC patients treated with IFN*α*, these thyroid disorders were well recognized as serious side effects. Previously, it was found that among CHC patients that received IFN therapy, elderly women were shown to have a 4.4 times higher risk of developing thyroid dysfunction than men [5]. Our findings were concordant with these previous findings. However, the detailed mechanism of IFN-induced thyroid autoimmunity remains unknown [19, 20].

Recently, BAFF was identified to be a 285-amino-acid protein that belongs to the TNF ligand superfamily [6–10]. After a serum BAFF assay was developed using a monoclonal antibody, clinical studies of several autoimmune diseases were conducted [11–15]. In particular, BAFF was found to be strongly associated with the emergence of several autoantibodies [16]. So, in the present study, we investigated the relationship between IFN-induced thyroid autoimmunity and serum BAFF.

First, the serum BAFF baseline levels before IFN therapy were significantly higher in group A than in group B. No difference was observed between group B and healthy control subjects. This result indicates that a high serum BAFF level before IFN therapy is a risk factor affecting the development of thyroid autoimmunity during IFN therapy. IFN*α* can cause a significant increase in anti-TPO levels in individuals who are positive for anti-TPO before IFN therapy [5]. Even in individuals in whom autoantibody tests were negative before IFN therapy, it was suggested that autoimmunity including thyroid disorders was amplified by IFN*α* therapy in patients showing high serum concentrations of BAFF before therapy.

Second, the mean serum BAFF levels detected at six months after the start of IFN therapy were significantly higher than those observed before therapy in both groups; that is, we found that serum BAFF levels were increased by IFN therapy.

Interestingly, there was a case undergoing type I IFN therapy developed RA, and this was also associated with increased levels of BAFF [21]. One potential consequence of high BAFF levels is the emergence of autoimmunity during IFN*α* therapy.

Finally, based on the hypothesis that BAFF might promote autoimmune diseases [22], clinical trials using BAFF inhibitors have been performed in RA and SLE patients [23]. These results could lead to the development of new strategies for treating IFN-induced thyroid autoimmunity.

## 5. Conclusion

Our findings suggest that the high values of serum BAFF concentration before IFN therapy can predict the risk of thyroid autoimmunity in elderly female patients with CHC.
